# Functional connectivity of animal-dispersed plant communities depends on the interacting effects of network specialization and resource diversity

**DOI:** 10.1098/rspb.2024.2995

**Published:** 2025-03-05

**Authors:** Anna R. Landim, Eike Lena Neuschulz, Isabel Donoso, Marjorie C. Sorensen, Thomas Mueller, Matthias Schleuning

**Affiliations:** ^1^ Senckenberg Biodiversity and Climate Research Centre (SBiK-F), Frankfurt am Main 60325, Germany; ^2^ Department of Biological Sciences, Goethe University Frankfurt, Frankfurt am Main 60438, Germany; ^3^ Basque Centre for Climate Change (BC3), Parque Científico UPV-EHU, Leioa 48940, Spain; ^4^ IKERBASQUE, Basque Foundation for Science, Bilbao 48009, Spain; ^5^ Instituto Mediterráneo de Estudios Avanzados (IMEDEA, CSIC-UIB), Esporles, Balearic Islands 07190, Spain; ^6^ Department of Biology, Kwantlen Polytechnic University, Surrey, British Columbia 12666 72 Ave, Canada

**Keywords:** plant–animal interactions, community assembly, forest recovery, restoration, seed dispersal, fruit tracking

## Abstract

Plant functional connectivity—the dispersal of plant propagules between habitat patches—is often ensured through animal movement. Yet, there is no quantitative framework to analyse how plant–animal interactions and the movement of seed dispersers influence community-level plant functional connectivity. We propose a trait-based framework to quantify plant connectivity with a model integrating plant–frugivore networks, animal-mediated seed-dispersal distances and the selection of target patches by seed dispersers. Using this framework, we estimated how network specialization, between-patch distance and resource diversity in a target patch affect the number and diversity of seeds dispersed to that patch. Specialized networks with a high degree of niche partitioning in plant–frugivore interactions reduced functional connectivity by limiting the diversity of seeds dispersed over long distances. Resource diversity in the target patch increased both seed number and diversity, especially in specialized networks and within short and intermediate distances between patches. Notably, resource diversity was particularly important at intermediate distances, where the number and diversity of seeds reaching a patch increased more strongly with resource diversity than at longer distances. Using a trait-based framework, we show that resource diversity in the target patch is a major driver of connectivity in animal-dispersed plant communities.

## 1. Introduction

Plant functional connectivity refers to the dispersal of plants and their genes between habitat patches [1,2]. High connectivity promotes the recolonization of habitat patches where plants have gone locally extinct and is essential to the restoration of plant communities [3,4]. For most plants, the movement underpinning this connectivity happens through seed dispersal by animals [5]. In the tropics, 75–90% of plant species depend on animals for seed dispersal [2,6]. Plant and animal traits shape the occurrence and frequency of frugivory and seed-dispersal interactions [7,8] and can be used to estimate distances of animal-mediated seed dispersal for entire plant communities (e.g. [9,10]). Despite the importance of animals for seed dispersal, there is still no comprehensive framework that allows a mechanistic understanding of how the combined effects of plant–animal interactions and animal movement shape animal-mediated plant functional connectivity [11,12]. Such a framework can be a valuable tool to reveal how plant–animal interactions and animal movement together drive connectivity and influence the restoration of plant communities.

Plant dispersal is intertwined with the movement ecology of their dispersal vectors [5] and can be separated into two components: the vector definition and the actual movement. In animal-mediated seed dispersal, the vector can be determined by an animal selecting certain fruits [13,14]. Plant dispersal, i.e. seed dispersal from point A to point B, depends on the distance and direction of the seed dispersing animal’s movement. Both movement distance and direction are often constrained by the animal’s motion capacity, which is usually related to its morphological traits [15,16]. Both also relate to navigation and the animals’ decision of where to target its movement [17,18]. This decision is influenced by the animal’s internal state, which drives movement based on specific goals, such as foraging movements to preferred resources [17,19]. Integrating movement and functional ecology into seed-dispersal studies (e.g. to estimate seed-dispersal distances as in [9,10]) can offer a deeper understanding of how plant–animal interactions and animal movements influence plant functional connectivity [20,21].

The vectors of seed dispersal can be identified through community-wide interaction networks that can be used to quantify how frequently animal species disperse the seeds of particular plant species. These networks represent species interactions, linking plants to their animal seed dispersers at the level of entire ecological communities. Within these networks, interaction probabilities between plants and animals depend on the compatibility of their traits, especially in the tropics [8,22]. Interaction probabilities are higher when plant and animal species traits closely match [14]. The degree of trait matching, e.g. the specificity of matching between fruit size and avian gape size, relates to the level of network specialization [23,24]. A high degree of trait matching reduces the number of potential interaction partners, leading to greater niche partitioning among seed dispersers and more exclusive interactions in specialized networks. In contrast, a low degree of trait matching results in more generalized networks, where most plant species share their seed dispersers [15,25]. Based on these trait-matching rules, models can be used to simulate seed-dispersal networks with different degrees of specialization.

Seed-dispersal distances may vary across different animal seed dispersers [26]. Dispersers’ traits that relate to movement ability and seed digestion may affect seed dispersal distances [11,27]. For instance, larger birds tend to retain seeds for a longer period of time and fly faster than smaller birds [28,29], dispersing seeds over longer distances [30]. By studying seed dispersal by different animal species, models can predict the cumulative distances of all potential seed dispersal events of a plant species that together form a seed-dispersal kernel [31–33]. In combination with interaction networks, trait-based movement models allow the estimation of total seed-dispersal kernels for entire plant communities [15]. Typically, these kernels show a higher density of dispersal events at shorter distances, with the probability of dispersal decreasing as distance increases [33,34]. Consequently, plant functional connectivity between habitat patches likely decreases with distance, as seeds have lower chances of reaching patches at longer distances. Previous simulations have shown that specialized networks result in shorter community-wide seed dispersal distances than generalized networks, suggesting that large birds that are essential for seed dispersal over long distances contribute less to seed dispersal of the entire plant community in specialized compared to generalized networks [15]. However, such effects of network specialization on seed-dispersal distances have not been tested in simulations of plant functional connectivity, which additionally depends on how seed dispersers direct their movement to potential target patches.

Movement direction is crucial for plant functional connectivity because animals can provide directed dispersal to specific locations [35,36]. An important, but rather little-studied determinant of movement direction is the resource diversity in a habitat patch, which is likely to influence the chances of seed dispersal to patches [37,38] because foraging is one of the primary motivations for frugivore movements [27]. Frugivores can track resources to minimize foraging time and maximize energy intake [39,40]. At a landscape scale, frugivorous birds are attracted to areas with greater fruit abundance and diversity, and, upon arrival, they tend to forage selectively on specific fruits [41,42]. Therefore, the movement of seed dispersers towards a target patch can be influenced by the abundance of their preferred fruit resources [43]. For example, larger birds that prefer larger fruits are more likely to occur in habitats with large fruits, while smaller birds are likely to favour patches with smaller fruits [44]. Thus, the resource diversity in a patch can be used to estimate the probability that frugivores will disperse seeds to that patch. Therefore, patches with a greater diversity of fruiting plants are likely to attract a broader range of animals [45], which is likely to promote community-level plant functional connectivity.

**Figure 1.**
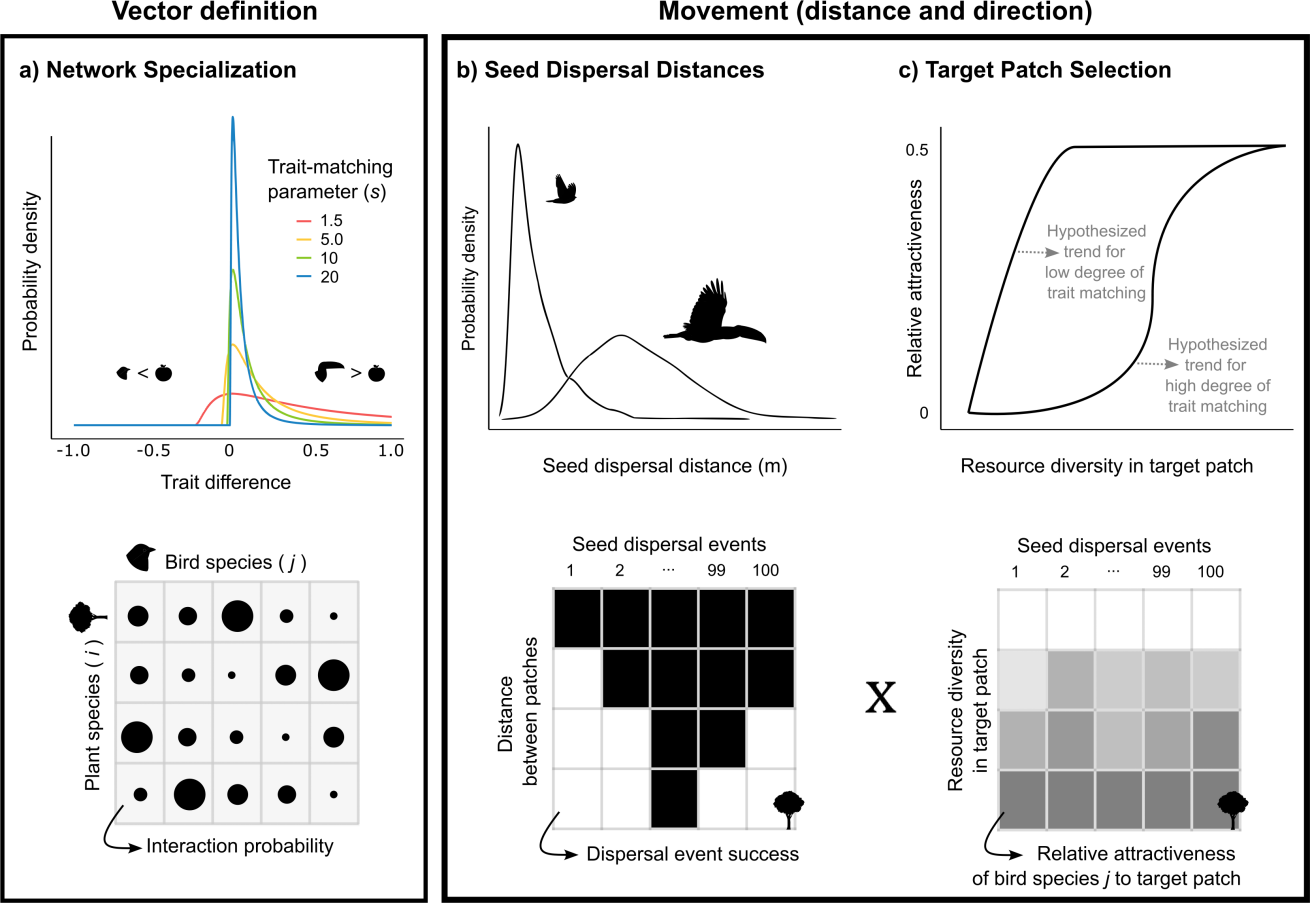
Quantitative framework for community-level plant functional connectivity. (a) The degree of trait matching between plants and birds defines network specialization in the source patch and the specific dispersal vectors of each plant species [25]. Interaction probabilities are represented by circle sizes in the resulting matrix; the degree of trait matching is varied by the parameter ‘*s*’ in the trait-matching function [24]. (b) Seed dispersal distances define reachable habitat patches. Total dispersal kernels for entire plant communities are simulated using avian traits [15]. In the resulting matrix, black squares indicate potentially successful dispersal events to a target patch. (c) Resource diversity in the target patch defines movement direction. Movement probability towards a target patch is quantified via its relative attractiveness. We hypothesized differences in the relative attractiveness of a target patch for low and high degrees of trait matching. At low trait matching, birds are attracted to many plant species, resulting in a gradual increase in relative attractiveness as resource diversity increases. At high trait matching, birds are attracted by only a few plant species, so relative attractiveness quickly increases once these species are present. In the resulting matrix, grey intensity corresponds to the relative attractiveness of a target patch for a specific bird species.

Here, we use a trait-based simulation model to investigate how the movement of avian seed dispersers influences plant functional connectivity across entire plant communities. We built upon established trait-based models and added two important landscape-level aspects (i.e. between-patch distance and resource diversity in the target patch) to these models to test how seed-disperser movement may affect functional connectivity at the plant community level. We focused on birds owing to their high mobility [46] and their crucial role as seed dispersers [47,48] and leveraged established trait-based models parameterized with bird data. With our model, we tested the previously unexplored interacting effects of network specialization, between-patch distance and resource diversity in the target patch on plant functional connectivity (figure 1). In the simulations, we quantified community-level plant functional connectivity by calculating both the number and the diversity of seeds reaching a target patch from a source patch. We hypothesized that plant functional connectivity decreases with increasing network specialization [15] and between-patch distance [49], and that it increases with resource diversity in a target patch. Importantly, we predicted that these three factors are not independent from each other, but interact in their effects on plant functional connectivity.

## 2. Methods

We present a quantitative framework to assess community-level plant functional connectivity through avian-mediated seed dispersal, building on existing models while integrating new elements. Specifically, we used established models for the first two components: (i) network specialization [25] and (ii) seed-dispersal distance [15]. The novelty of our framework lies primarily in the third component: (iii) resource diversity in the target patch (figure 1c). To construct our model, we simulated seed-dispersal networks for a community composed of 60 bird and 50 plant species, using trait information from empirical plant and bird communities collected along the Manú elevational gradient in Peru [25] (see electronic supplementary materials, Methods for details). Previous studies have shown that the trait distributions found in these tropical forests are also representative of other tropical plant and bird communities [8,50]. We opted for this simulation approach to allow greater flexibility to explore the effects of our model components. However, our framework can also be applied to empirical interaction networks.

Our framework comprises three components of seed dispersal to estimate community-level plant functional connectivity. First, we varied the degree of trait matching to simulate networks in the source patch that varied in their degree of network specialization (figure 1a). A high degree of trait matching results in specialized networks and a low degree of trait matching results in more generalized networks [15]. Second, we simulated seed dispersal events for all plant species in the network, yielding a total seed-dispersal kernel for the entire plant community. For each seed-dispersal event (figure 1b), we estimated the seed-dispersal distances using a trait-based model [15] and then calculated the probability of successful seed dispersal events to target patches with different distances to the source patch. Third, we quantified the relative attractiveness of the target patch, based on its resource diversity (richness and abundance) relative to the source patch (figure 1c). We hypothesized that relative attractiveness would gradually increase if trait matching is low, because birds would be attracted by more plant species in the community. In contrast, high degrees of trait matching constrain birds to feed on specific plant species and attractiveness would then depend on the presence of these specific species (as illustrated in figure 1c). Finally, to combine the results from the simulations of seed-dispersal distance and direction, we multiplied the probability of seeds reaching a target patch (distance) and the relative attractiveness of a target patch (direction) to estimate both the number and the effective diversity of seeds dispersed to the target patch.

### (a) Network specialization

The first step of our framework relates to the definition of dispersal vectors, identifying which bird species are responsible for the dispersal of each plant species in the source patch. By varying the degree of trait-matching (*s* [24]), we simulated networks with different degrees of network specialization (*H*_2_′ [51] at the source patch, ranging from generalized (*s* = 1.5, *H*_2_′ = 0.16) to specialized (*s* = 20, *H*_2_′ = 0.65) networks (figure 1a); comparable with the variation in the specialization of empirical seed-dispersal networks [52]. These simulations were done to test our hypothesis that plant functional connectivity decreases with network specialization.

The seed-dispersal network of the source patch was simulated using trait matching between plants and birds [10,15,25]. Interaction probabilities were estimated by multiplying the probabilities of interactions derived from two pairs of matching traits. Fruit and gape size provide information about a bird’s preferred fruit selection, e.g. birds with larger gapes tend to eat larger fruits [14]. Plant height and wing pointedness (standardized to the same scale) determine the forest strata primarily used, e.g. birds with pointier wings tend to prefer to feed on taller trees [8]. For the matching between fruit and gape size, we employed a right-skewed niche shape [25]. That is, a negative mismatch, i.e. when fruit size is larger than the gape size (forbidden interactions; [53]), leads to a rapid decrease in interaction probability, whereas a positive mismatch leads to a more gradual decrease in interaction probability ([14]; figure 1a). For the relationship between plant height and wing pointedness, we used a Gaussian distribution because the probability of interactions decreases symmetrically for both negative and positive mismatches [8].

The interaction probabilities from our simulated seed-dispersal networks were weighted by the abundances of both bird and plant species. Abundances were estimated by allometric relationships based on fruit size and fruit abundance, as well as bird size and abundance, assuming that small fruits and small birds are more abundant than larger ones and thus are more likely to interact (following [25]; see electronic supplementary material, Methods for details). To obtain accurate estimates for each plant species, we simulated 100 dispersal events for each plant species in the community. We also tested an alternative scenario in which dispersal events were weighted by plant species abundances, assuming that small-fruited and therefore more abundant plant species had more dispersal events. These additional simulations yielded very similar results (see electronic supplementary material, figures S1 and S2).

### (b) Seed-dispersal distance

The second step of our framework relates to the transfer of seeds from the source to the target patch. We estimated the distance of seed dispersal events and calculated dispersal success depending on the distance between source and target patches (figure 1b). This involved testing our hypothesis that plant functional connectivity decreases with distance between patches. We varied the distances in 10 m intervals from 0 to 600 m—approximately 30 m beyond the maximal distance predicted by our seed-dispersal distance simulations (see electronic supplementary material, figures S3 and S4).

To simulate seed-dispersal distances, we used a trait-based model that relies on bird body mass [15]. Gut passage time was estimated based on bird body mass, using estimates derived from frugivorous birds' behaviour in natural environments (e.g. [18,26,54]). Then, using information from empirical data, flight speed was estimated as a function of bird body mass [15]. The combination of gut passage time and flight speed was used to estimate dispersal distances. A calibration term was incorporated to take into account time spent not moving and deviations from linear flight paths (for details on these calculations see the electronic supplementary material, Methods).

### (c) Resource diversity in the target patch

The third and final step of our framework relates to the selection of a target patch by the animal seed dispersers. To test the hypothesis that resource diversity in the target patch increases plant functional connectivity, we introduce a metric named *relative attractiveness*, which estimates the probability that birds visit the target patch based on resource diversity (figure 1c). This metric compares a bird’s attraction with the fruit resources (i.e. the abundance and richness of fruits) in the target patch relative to those present in the source patch. Fruit resource abundance was estimated based on plant species-specific fruit mass, assuming that smaller fruits are more abundant than large ones [55]. In our simulations, the source patch contained the complete plant community (50 species with their respective fruit abundances), while we varied the resource diversity in the target patch by incrementally increasing the number of plant species from 0 to 50. This was repeated over 100 iterations so that species composition changed at each richness level. Given that each plant species produced a fixed number of fruits in our model, these simulations also varied resource abundance in the target patch. To account for the fact that we simultaneously varied both resource richness and abundance, we report resource diversity as the effective number of fruit species present in a community (i.e. the exponential of Shannon diversity, which represents the number of equally common species [56]).

Our model first estimated the *general attractiveness* (*A*) of a patch in terms of its resource diversity and the bird’s attraction to this patch. General attractiveness was quantified based on trait matching and resource abundance. A higher degree of trait matching results in an increased interaction probability to a few specific plant species, making a patch more attractive if it contains those species with a high fruit abundance. In scenarios with a low degree of trait matching, the attractiveness of a patch is less dependent on the availability of specific fruit resources. In summary, the general attractiveness of a patch (*k*) to a bird (*j*) was quantified as


$$A_{k}^{j}=\Sigma_{i\;\in patch\;k\;}P_{i,j}^{\prime }\;b_{i},$$

where $A_{k}^{j}$ is the general attractiveness of patch *k* to bird *j*, *P'_i,j_* is the probability of interaction between bird *j* and plant *i* and *b_i_* is the abundance of plant *i*.

We propose that a bird’s visiting probability depends on the attractiveness of the target relative to the source patch. Relative attractiveness (*RA*) is then defined as


$$RA^{j}=\frac{A_{recipient}^{j}}{A_{recipient}^{j}+\;A_{source}^{j}},$$

representing the probability of bird *j* visiting the target patch. When the target patch has no resources, its attractiveness and relative attractiveness are zero for all bird species. When both patches have identical plant communities, relative attractiveness is 0.5, which represents the maximum value in our simulations because the source patch always contained the complete plant community. By running 100 iterations with varying species richness and composition, we tested the effect of resource diversity on plant functional connectivity.

We conducted an additional set of simulations excluding the interaction probability between two species (*P'_i,j_*) from this part of the model, which enabled us to remove the effect of trait matching and to solely test the effect of resource abundance in the target patch on plant functional connectivity.

### (d) Community-level plant functional connectivity

We integrated the three components of our model to estimate community-level plant functional connectivity. To this end, we calculated the probability of seeds from each plant species in the source patch community reaching a target patch and, based on this, we quantified both the number and diversity of seeds per species arriving in the target patch. We used both measures to compare plant functional connectivity under different combinations of network specialization, distance between patches and resource diversity in the target patch.

For each level of network specialization (figure 1a), we estimated the probability of seeds reaching the target patch across all simulated between-patch distances and resource diversity scenarios. This was done by multiplying the probability matrices related to seed-dispersal distance (seed-dispersal distance relative to distance between patches, figure 1b) and target patch selection (attractiveness of a target patch relative to the source patch, figure 1c). The result was a new matrix representing the probability of seeds reaching the target patch for each plant species. To obtain community-level measures, we then summed these probabilities across all dispersal events of each plant species to calculate the potential number of seeds reaching the target patch in each scenario. For example, if all 100 seed-dispersal events of a plant species had a 0.5 probability under a specific combination of between-patch distance and resource diversity, we considered that 50 seeds would reach the target patch. If the sum was below 1, we set the number of seeds to 0, indicating that no seeds from that plant species would reach the target patch in that scenario. To estimate the diversity of seeds arriving at a target patch, we applied the Shannon diversity index to the probabilities of all plant species. Seed diversity was then transformed into the effective seed diversity by taking the exponential of the Shannon diversity index [56].

## 3. Results

Our simulations generated seed-dispersal distances ranging from 0.4 to 555 m. We focused on the range from 10 to 250 m because we considered distances lower than 10 m too short for between-patch seed dispersal, and the chances of seed dispersal beyond 250 m were very low (see electronic supplementary material, Results, figures S1 and S2). The number and diversity of seeds reaching the target patch were used as two alternative measures of plant functional connectivity and they showed distinct responses to the effects of network specialization, between-patch distance and resource diversity in the target patch.

**Figure 2.**
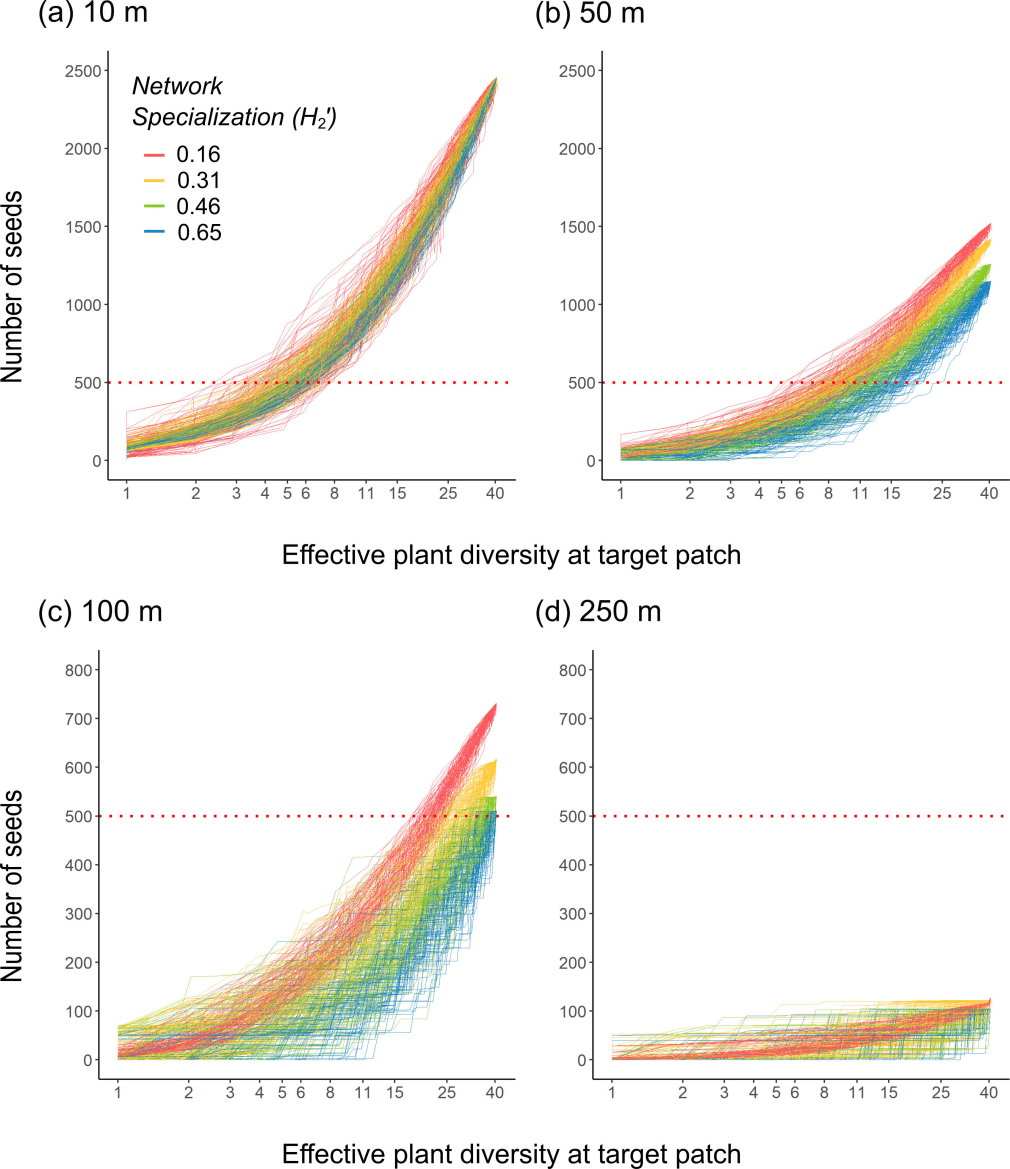
The combined effects of network specialization, between-patch distance and resource diversity in the target patch on the number of seeds that reach the target patch. The effective plant diversity in the target patch (shown on a log-scale) defines the attractiveness of a target patch relative to the source patch comprising an effective diversity of 40 plant species. Different colours indicate different levels of network specialization and each line represents one of the 100 iterations across different values of effective plant diversity in the target patch. The number of seeds is plotted in relation to network specialization level and resource diversity for (a) 10 m, (b) 50 m, (c) 100 m and (d) 250 m distances between patches. Please note that the red dotted line indicates a difference in the scale of the number of dispersed seeds (*y*-axis) from (a) and (b) to (c) and (d).

The simulations showed that the number of seeds reaching a target patch varied little across different levels of network specialization (figure 2). As expected, the number of seeds decreased rapidly with increasing distance between patches. According to the simulations, up to 2500 seeds reached the target patch at a distance of 10 m, while this number was reduced to about 125 seeds at 250 m distance. The number of dispersed seeds was also affected by the resource diversity in the target patch. Most seeds reached the target patch when resource diversity was at its maximum effective diversity of 40 plant species. The positive effect of resource diversity on the number of seeds reaching the target patch was most pronounced at intermediate distances between patches (figure 2). As distance increased, a greater resource diversity in the target patch was required to maintain a high plant functional connectivity in terms of seed numbers. For example, while 500 seeds reached the target patch at a 10 m distance with only six species present, achieving the same number at 100 m required between 25 and 40 plant species (contingent on the level of network specialization). However, at 250 m, the number was lower than 100 seeds across all values of resource diversity.

**Figure 3.**
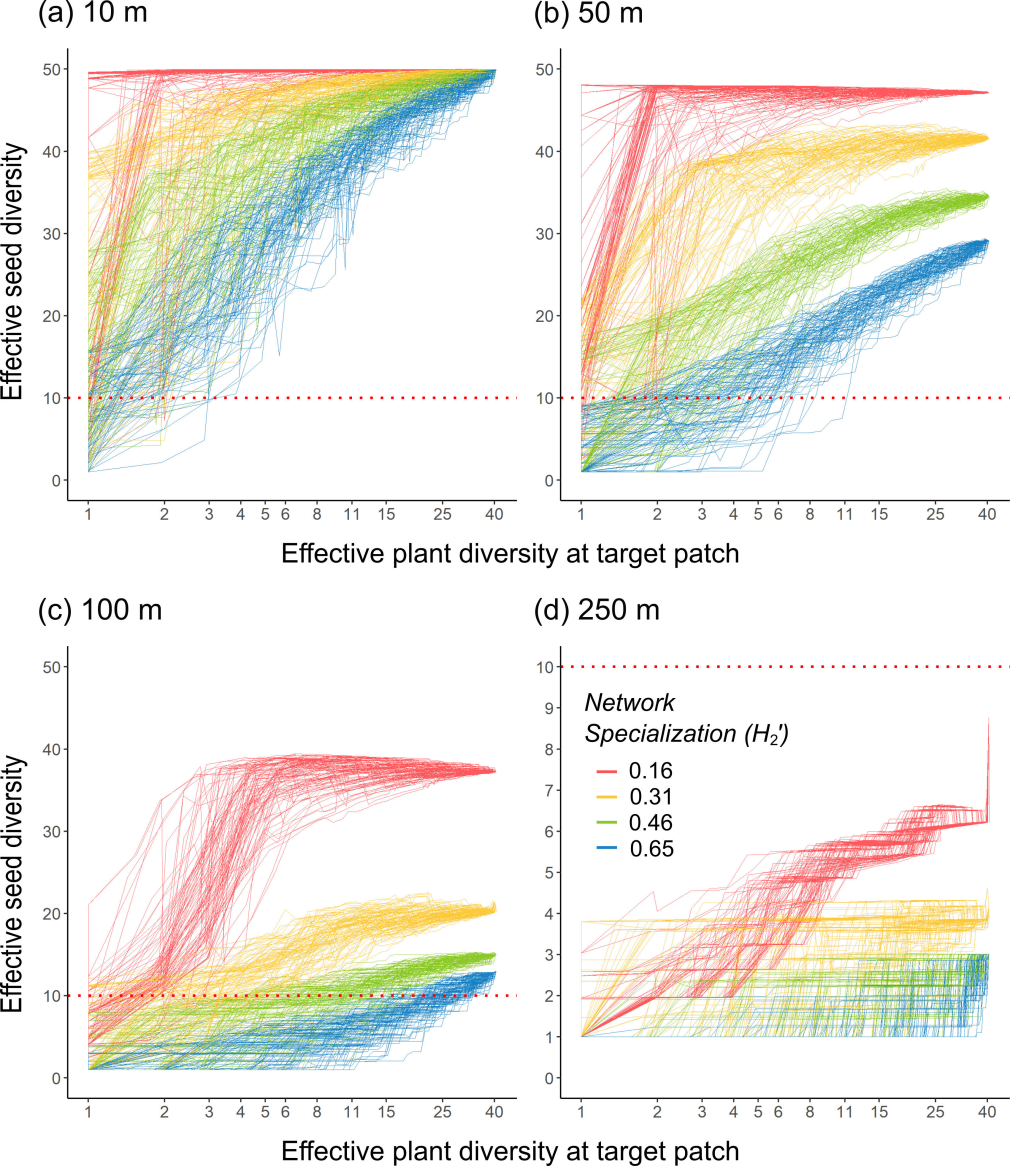
Shown are the combined effects of network specialization, between-patch distance and resource diversity in the target patch on the diversity of seeds that reach the target patch. The effective plant diversity in the target patch (shown on a log-scale) defines the attractiveness of a target patch relative to the source patch comprising an effective diversity of 40 plant species. Different colours indicate different levels of network specialization and each line represents one of the 100 iterations across different values of effective plant diversity in the target patch. The effective diversity of seeds is plotted in relation to network specialization and resource diversity in the target patch for (a) 10 m, (b) 50 m, (c) 100 m and (d) 250 m distances between patches. Please note that in (d), the scale of the effective diversity of dispersed seeds (*y*-axis) was adjusted as indicated by the red dashed line.

In the simulation models, the effective diversity of seeds reaching the target patch was affected by the interacting effects of network specialization, between-patch distance and resource diversity (figure 3). The simulations showed that generalized networks generally provided a greater diversity of seeds and, thus, a higher plant functional connectivity compared with specialized networks. For instance, at a distance of 100 m between patches, the effective diversity of seeds was below 20 plant species in the most specialized networks, whereas it was above 30 plant species in the most generalized network (figure 3). The effective diversity of seeds rapidly decreased with increasing distance, particularly in specialized networks. Conversely, resource diversity increased the diversity of seeds reaching the target patch. The presence of a diverse plant community in the target patch was most important in specialized networks and at intermediate distances. At the shortest distance of 10 m, an effective diversity of five plant species in the target patch was sufficient to maintain an effective seed diversity of at least 25 plant species even for the most specialized network. At 50 m, such effective seed diversity in the target patch was only achieved for the same level of specialization if the target patch had its maximum resource diversity. At 250 m, the effective diversity of seeds reaching the target patch was only about six species in the most generalized network and for maximum resource diversity.

In simulations excluding the effect of trait matching to estimate patch attractiveness (i.e. to understand the effect of resource abundance alone), we found similar patterns for the number of seeds reaching a target patch (electronic supplementary material, figure S5). However, trait matching had a stronger effect on the diversity of seeds and led to more pronounced effects of network specialization and resource diversity on seed diversity (electronic supplementary material, figure S6).

## 4. Discussion

We developed a quantitative framework to analyse the interacting effects of network specialization, between-patch distance and resource diversity in the target patch on community-level plant functional connectivity. We found that resource diversity in the target patch generally increased both the number and diversity of dispersed seeds, but that this effect changed with the level of network specialization and distance between patches. Particularly, the effect of resource diversity on connectivity was most pronounced at high levels of specialization and at intermediate distances between patches. Overall, our simulations suggest that diverse plant communities can attract a wider range of seed-dispersing bird species, which is crucial for maintaining high functional connectivity and for promoting forest restoration.

In our simulations, we found that lower network specialization increased the diversity of seeds reaching the target patch. While the number of seeds remained almost constant across different levels of specialization, birds in less specialized networks dispersed a more diverse set of plant species to the target patch compared with those in specialized networks (see also [15]). For generalized networks, we found that even when the resource diversity in the target patch was low or the distance between patches was large, a high diversity of plant species was still dispersed. This raises the chances of successful recruitment and a rapid increase in plant diversity in these patches. In contrast, in specialized networks, this process might be slower owing to a more limited diversity of seeds dispersed to the target patch. Interestingly, the decrease in connectivity with increasing specialization varied with distance. At shorter distances between patches (10 and 50 m), connectivity gradually decreased with specialization level, whereas at larger distances (>100 m), this effect became more pronounced and we observed a sharp decline in connectivity at specialization values larger than 0.3. In real-world networks, specialization levels typically range between 0.2 and 0.5 [52], suggesting that the low levels of connectivity at distances larger than 100 m may be representative of natural conditions.

Our findings also align with previous empirical works that show that generalist bird species play a key role in enhancing plant diversity and habitat connectivity, which benefits plant regeneration in early successional forests [48,57]. While existing studies mostly focus on the dichotomy between forest and matrix habitats [12,58], our simulation quantifies how the specificity of plant–bird interactions may influence the diversity of seeds that can be dispersed between habitat patches. This extends previous empirical work, which has shown that specialized networks can provide higher seed density [58]. Our framework could also be valuable to explore metacommunity dynamics [59], offering a tool to test how interaction specificity (either through preference or specialization [60]), shapes functional connectivity across fragmented landscapes. Furthermore, based on empirical studies of network specialization along large- and small-scale environmental gradients, it may be possible to infer through our simulations that plant functional connectivity may be greater in temperate rather than tropical climates and in the Afrotropics rather than the Neotropics [61,62]. Importantly, generalized plant–frugivore networks at forest edges may help to promote functional connectivity at the landscape scale [63].

The distance between habitat patches primarily affected the number of seeds dispersed to a target patch and affected seed diversity mostly indirectly via a reduction in seed numbers. The number of seeds arriving at a target patch strongly depended on the resource diversity in the target patch. According to our simulations, the number of seeds dropped significantly even at a between-patch distance of just 10 m if the resource diversity in the target patch was lower than 15 plant species. This highlights the importance of resource diversity for promoting seed dispersal between habitat patches. Because we found similar patterns at 0 and 10 m distances of seed dispersal (electronic supplementary material, figures S3 and S4), similar effects may be expected for seed dispersal within habitat patches. This suggests that seed dispersal within patches is most likely in patches with a high resource diversity. This is important because seed dispersal within patches contributes to plant regeneration and population persistence [64].

The findings of our simulations are consistent with numerous empirical [30,49,65] and simulation studies [10,54,66] of avian seed dispersal. For instance, Camargo *et al*. [49] reported a significant reduction in seed density from patches located at 10–50 m distance to forest remnants and recorded almost no seeds reaching plots at 300 m distance from the forest source. This is in line with our findings; however, rare long-distance seed dispersal events can be important for plant dispersal to more distant patches and to initiate the recolonization of remote sites [34,67]. Although these events can be crucial for plant populations, they may have little effect on the overall number and diversity of seeds dispersed at the community level.

Previous work suggested that short-distance seed dispersal is most relevant for community-level plant functional connectivity and that habitat patches may be crucial as stepping stones in real-world landscapes [64,68]. Our simulations suggest that a high patch density may be required to enhance the functional connectivity of bird-dispersed plant communities because most dispersal events are likely to happen between neighbouring patches. Nevertheless, we caution against an overinterpretation of the specific seed-dispersal distances predicted by our trait-based model because the absolute distances may vary depending on the assumptions of the model and the trait distributions of plant and bird communities. Applying the framework to real-world data is therefore required to validate the prediction of seed-dispersal distances and identify potential distance thresholds for plant functional connectivity. Such empirical studies could also serve to test the relationship between resource diversity and the diversity of seeds arriving at habitat patches of different size (see [69] for a simulation of seed dispersal in a real landscape).

Resource diversity affected both the number and diversity of seeds reaching target patches in our simulations. In particular, in specialized networks and at intermediate distances between patches (50–100 m), a large resource diversity was required to sustain plant functional connectivity because the birds that disperse seeds over these distances are more selective towards their resources in specialized networks. These results align with previous studies showing that resource diversity becomes increasingly important with high levels of specialization [45,70]. At long distances (250 m or more), resource diversity of the target patch had little effect because the number and diversity of seeds that were predicted to be dispersed to long distances were so low that it was largely independent of resource diversity. This suggests that resource diversity plays a prominent role in maintaining plant functional connectivity in landscapes with a high patch density, whereas in more fragmented landscapes, connectivity primarily relies on rare long-distance dispersal events performed by large birds. It is therefore likely that large-fruited plants dispersed by large birds may have better gene flow in highly fragmented landscapes [71].

Our proposed framework resonates with recent seed-dispersal research that aimed to integrate concepts of fruit tracking and animal movement [27,72] and emphasized the importance of seed-disperser behaviour and seed-dispersal movements for plant community dynamics [5,21,69]. While many studies highlight the role of fruit abundance as a key driver of frugivore movements [37,38,47,73], avian frugivores’ preferential selection of specific fruits [60] is also a key driver of their movement [41,74]. Our additional analysis, which excludes trait-matching from the calculation of relative attractiveness, shows that resource abundance seems to be the main driver of the number of dispersed seeds [75]. However, our simulations also demonstrate that plant–animal trait-matching is particularly important in determining the diversity of seeds dispersed into habitat patches. More specifically, we show that plant functional connectivity may critically depend on the diversity of available fruit resources and how these fruits are selected by the seed dispersers. High resource diversity in a target patch may therefore also promote the potential for directed dispersal into such patches [76,77].

By showing the importance of resource diversity, our simulations have important implications for ecological restoration. We found that the functional connectivity of animal-dispersed plant communities critically depends on the diversity of both bird and plant species, and that it can be increased by attracting a diverse set of seed-dispersing bird species to regenerating habitat patches. Active restoration measures, such as planting fruiting trees and shrubs, may therefore significantly assist the regeneration process and help direct animal seed dispersers to regenerating forest ‘islands’ [77–79], especially if plant diversity in such islands is high [80]. In line with our findings, the inclusion of keystone resources—plants with fruits that are highly attractive to different groups of seed dispersers—is likely to increase the dispersal of seeds into regenerating habitat patches [81,82]. Additionally, planting ‘little islands’ of fruit resources could improve connectivity in highly fragmented landscapes, accelerating forest restoration [69,83]. While our framework focuses on the morphological traits of the plant community, it is important to acknowledge that other traits, such as the nutritional content of the fruits or phenological variation among plant species, significantly affect foraging decisions by frugivores [42,74,84] and may influence the plant species reaching target patches [49]. Future studies on plant functional connectivity should also integrate other types of fruit traits to provide a more comprehensive understanding of the mechanisms driving plant functional connectivity. Moreover, other factors such as forest cover and patch size are known to play important roles in restoration dynamics [69,85]. While these factors may be crucial to explain the context-dependence of restoration dynamics [86,87], our simulations suggest that a high fruit diversity, which is likely to be positively related to patch size [88], may generally enhance plant functional connectivity. Another important extension in future simulation model studies should be the inclusion of other animal seed dispersers such as frugivorous bats, as these species are often particularly important at early stages of forest recovery [89,90].

By proposing a trait-based framework to assess the functional connectivity of bird-dispersed plant communities, we integrate connectivity concepts from plant and movement ecology. We foresee that the application of our quantitative framework, which comprises all major processes of seed dispersal by animals, can contribute to a more mechanistic understanding of the processes that limit plant functional connectivity in real-world landscapes. The increasing availability of plant and bird trait data [91,92] will make it rather easy to apply the framework to different types of study systems, as this would only require the availability of plant and bird community data for a mosaic of habitat patches. Applying the framework to real-world data can yield a quantitative understanding of plant functional connectivity in different types of landscapes and ecosystems, as well as help to provide practical guidance to forest restoration projects.

## Data Availability

The data used are already publicly available at [93]. The dataset and code supporting this study are available at Zenodo [94]. Supplementary material is available online [95].

## References

[1] Tischendorf L, Fahrig L. 2000 On the usage and measurement of landscape connectivity. Oikos **90**, 7–19. (10.1034/j.1600-0706.2000.900102.x)

[2] Rogers HS, Donoso I, Traveset A, Fricke EC. 2021 Cascading impacts of seed disperser loss on plant communities and ecosystems. Annu. Rev. Ecol. Evol. Syst. **52**, 641–666. (10.1146/annurev-ecolsys-012221-111742)

[3] Wunderle JM. 1997 The role of animal seed dispersal in accelerating native forest regeneration on degraded tropical lands. For. Ecol. Manage. **99**, 223–235. (10.1016/S0378-1127(97)00208-9)

[4] Aavik T, Helm A. 2018 Restoration of plant species and genetic diversity depends on landscape‐scale dispersal. Restor. Ecol. **26** S92–S102. (10.1111/rec.12634)

[5] Damschen EI, Brudvig LA, Haddad NM, Levey DJ, Orrock JL, Tewksbury JJ. 2008 The movement ecology and dynamics of plant communities in fragmented landscapes. Proc Natl. Acad. Sci. USA **105**, 19078–19083. (10.1073/pnas.0802037105) 19060187 PMC2614718

[6] Howe HF, Smallwood J. 1982 Ecology of seed dispersal. Annu. Rev. Ecol. Syst. **13**, 201–228. (10.1146/annurev.es.13.110182.001221)

[7] Schleuning M, Fründ J, García D. 2015 Predicting ecosystem functions from biodiversity and mutualistic networks: an extension of trait‐based concepts to plant–animal interactions. Ecography **38**, 380–392. (10.1111/ecog.00983)

[8] Bender IMA *et al*. 2018 Morphological trait matching shapes plant–frugivore networks across the Andes. Ecography **41**, 1910–1919. (10.1111/ecog.03396)

[9] Donoso I, Sorensen MC, Blendinger PG, Kissling WD, Neuschulz EL, Mueller T, Schleuning M. 2020 Downsizing of animal communities triggers stronger functional than structural decay in seed-dispersal networks. Nat. Commun. **11**, 1582. (10.1038/s41467-020-15438-y) 32221279 PMC7101352

[10] Nowak L *et al*. 2022 Avian seed dispersal may be insufficient for plants to track future temperature change on tropical mountains. Glob. Ecol. Biogeogr. **31**, 848–860. (10.1111/geb.13456)

[11] Auffret AG *et al*. 2017 Plant functional connectivity – integrating landscape structure and effective dispersal. J. Ecol. **105**, 1648–1656. (10.1111/1365-2745.12742)

[12] González-Varo JP *et al*. 2023 Frugivore-mediated seed dispersal in fragmented landscapes: compositional and functional turnover from forest to matrix. Proc. Natl Acad. Sci. **120**, e2302440120. (10.1073/pnas.2302440120) 37871198 PMC10622928

[13] González-Castro A, Yang S, Nogales M, Carlo TA. 2015 Relative importance of phenotypic trait matching and species’ abundances in determining plant–avian seed dispersal interactions in a small insular community. AoB Plants **7**, v017. (10.1093/aobpla/plv017) PMC4372831 25750409

[14] Dehling DM, Jordano P, Schaefer HM, Böhning-Gaese K, Schleuning M. 2016 Morphology predicts species’ functional roles and their degree of specialization in plant–frugivore interactions. Proc. R. Soc. B **283**, 7. (10.1098/rspb.2015.2444) PMC4795026 26817779

[15] Sorensen MC, Donoso I, Neuschulz EL, Schleuning M, Mueller T. 2020 Community‐wide seed dispersal distances peak at low levels of specialisation in size‐structured networks. Oikos **129**, 1727–1738. (10.1111/oik.07337)

[16] Zhang J, Pannell JL, Case BS, Hinchliffe G, Stanley MC, Buckley HL. 2021 Interactions between landscape structure and bird mobility traits affect the connectivity of agroecosystem networks. Ecol. Indic. **129**, 107962. (10.1016/j.ecolind.2021.107962)

[17] Nathan R, Getz WM, Revilla E, Holyoak M, Kadmon R, Saltz D, Smouse PE. 2008 A movement ecology paradigm for unifying organismal movement research. Proc. Natl. Acad. Sci. **105**, 19052–19059. (10.1073/pnas.0800375105) 19060196 PMC2614714

[18] Morales JM, García D, Martínez D, Rodriguez-Pérez J, Herrera JM. 2013 Frugivore behavioural details matter for seed dispersal: a multi-species model for cantabrian thrushes and trees. PLoS One **8**, e65216. (10.1371/journal.pone.0065216) 23776452 PMC3679117

[19] Abrahms B, Aikens EO, Armstrong JB, Deacy WW, Kauffman MJ, Merkle JA. 2021 Emerging perspectives on resource tracking and animal movement ecology. Trends Ecol. Evol. **36**, 308–320. (10.1016/j.tree.2020.10.018) 33229137

[20] Mueller T, Lenz J, Caprano T, Fiedler W, Böhning‐Gaese K. 2014 Large frugivorous birds facilitate functional connectivity of fragmented landscapes. J. Appl. Ecol. **51**, 684–692. (10.1111/1365-2664.12247)

[21] Graf V, Müller T, Grüebler MU, Kormann UG, Albrecht J, Hertel AG, Sorensen MC, Tschumi M, Neuschulz EL. 2024 Individual behaviour shapes patterns of bird‐mediated seed dispersal. Funct. Ecol. **38**, 1032–1043. (10.1111/1365-2435.14556)

[22] McFadden IR, Fritz SA, Zimmermann NE, Pellissier L, Kissling WD, Tobias JA, Schleuning M, Graham CH. 2022 Global plant‐frugivore trait matching is shaped by climate and biogeographic history. Ecol. Lett. **25**, 686–696. (10.1111/ele.13890) 35199916 PMC9302656

[23] Vázquez DP, Blüthgen N, Cagnolo L, Chacoff NP. 2009 Uniting pattern and process in plant–animal mutualistic networks: a review. Ann. Bot. **103**, 1445–1457. (10.1093/aob/mcp057) 19304996 PMC2701748

[24] Fründ J, McCann KS, Williams NM. 2016 Sampling bias is a challenge for quantifying specialization and network structure: lessons from a quantitative niche model. Oikos **125**, 502–513. (10.1111/oik.02256)

[25] Donoso I, Schleuning M, García D, Fründ J. 2017 Defaunation effects on plant recruitment depend on size matching and size trade-offs in seed-dispersal networks. Proc. R. Soc. B **284**, 20162664. (10.1098/rspb.2016.2664) PMC5454253 28566481

[26] Spiegel O, Nathan R. 2007 Incorporating dispersal distance into the disperser effectiveness framework: frugivorous birds provide complementary dispersal to plants in a patchy environment. Ecol. Lett. **10**, 718–728. (10.1111/j.1461-0248.2007.01062.x) 17594427

[27] Borah B, Beckman NG. 2022 Studying seed dispersal through the lens of movement ecology. Oikos **2022**, e08310. (10.1111/oik.08310)

[28] Robbins CT. 1993 Wildlife feeding and nutrition, 2nd edn. New York, NY: Academic Press.

[29] Godínez‐Alvarez H, Ríos‐Casanova L, Peco B. 2020 Are large frugivorous birds better seed dispersers than medium‐ and small‐sized ones? Effect of body mass on seed dispersal effectiveness. Ecol. Evol. **10**, 6136–6143. (10.1002/ece3.6285) 32607219 PMC7319144

[30] Jordano P, García C, Godoy JA, García-Castaño JL. 2007 Differential contribution of frugivores to complex seed dispersal patterns. Proc. Natl Acad. Sci. USA **104**, 3278–3282. (10.1073/pnas.0606793104) 17360638 PMC1805555

[31] Nathan R. 2007 Total dispersal kernels and the evaluation of diversity and similarity in complex dispersal systems. In Frugivory and seed dispersal: theory and its application in a changing world (eds AJ Dennis, EW Schupp, RJ Green, DA Westcott), pp. 252–276. Wallingford, UK: CAB International Publishing. (10.1079/9781845931650.0252)

[32] Klein EK, Robledo-Arnuncio JJ, Revilla E. 2012 Dispersal kernerls: review. In Dispersal ecology and evolution (eds J Clobert, JM Bullock), pp. 187–210. Oxford, UK: Oxford University Press.

[33] Rogers HS *et al*. 2019 The total dispersal kernel: a review and future directions. AoB Plants **11**, plz042. (10.1093/aobpla/plz042) 31579119 PMC6757349

[34] García C, Borda‐de‐Água L. 2017 Extended dispersal kernels in a changing world: insights from statistics of extremes. J. Ecol. **105**, 63–74. (10.1111/1365-2745.12685)

[35] Wenny DG, Levey DJ. 1998 Directed seed dispersal by bellbirds in a tropical cloud forest. Proc. Natl Acad. Sci. USA **95**, 6204–6207. (10.1073/pnas.95.11.6204) 9600942 PMC27627

[36] Wenny DG. 2001 Advantages of seed dispersal: a re-evaluation of directed dispersal. Evol. Ecol. Res. **3**, 37–50.

[37] Lehouck V, Spanhove T, Vangestel C, Cordeiro NJ, Lens L. 2009 Does landscape structure affect resource tracking by avian frugivores in a fragmented Afrotropical forest? Ecography **32**, 789–799. (10.1111/j.1600-0587.2009.05666.x)

[38] García D, Zamora R, Amico GC. 2011 The spatial scale of plant–animal interactions: effects of resource availability and habitat structure. Ecol. Monogr. **81**, 103–121. (10.1890/10-0470.1)

[39] Rey PJ. 1995 Spatio‐temporal variation in fruit and frugivorous bird abundance in olive orchards. Ecology **76**, 1625–1635. (10.2307/1938163)

[40] Saracco JF, Collazo JA, Groom MJ. 2004 How do frugivores track resources? Insights from spatial analyses of bird foraging in a tropical forest. Oecologia **139**, 235–245. (10.1007/s00442-004-1493-7) 14872335

[41] Blendinger PG *et al*. 2012 Fine‐tuning the fruit‐tracking hypothesis: spatiotemporal links between fruit availability and fruit consumption by birds in Andean mountain forests. J. Anim. Ecol. **81**, 1298–1310. (10.1111/j.1365-2656.2012.02011.x) 22742825

[42] Carlo TA *et al*. 2024 Negative density dependence characterizes mutualistic interactions between birds and fruiting plants across latitudes. Phil. Trans. R. Soc. B **379**, 20230128. (10.1098/rstb.2023.0128) 38913067 PMC11529629

[43] Donoso I, García D, Martínez D, Tylianakis JM, Stouffer DB. 2017 Complementary effects of species abundances and ecological neighborhood on the occurrence of fruit-frugivore interactions. Front. Ecol. Evol **5**, 1–12. (10.3389/fevo.2017.00133)

[44] Vollstädt MGR, Ferger SW, Hemp A, Howell KM, Töpfer T, Böhning‐Gaese K, Schleuning M. 2017 Direct and indirect effects of climate, human disturbance and plant traits on avian functional diversity. Glob. Ecol. Biogeogr. **26**, 963–972. (10.1111/geb.12606)

[45] Kissling WD, Rahbek C, Böhning-Gaese K. 2007 Food plant diversity as broad-scale determinant of avian frugivore richness. Proc. R. Soc. B **274**, 799–808. (10.1098/rspb.2006.0311) PMC2093978 17251107

[46] González‐Varo JP, Carvalho CS, Arroyo JM, Jordano P. 2017 Unravelling seed dispersal through fragmented landscapes: frugivore species operate unevenly as mobile links. Mol. Ecol. **26**, 4309–4321. (10.1111/mec.14181) 28503829

[47] Garcia D, Zamora R, Amico GC. 2010 Birds as suppliers of seed dispersal in temperate ecosystems: conservation guidelines from real‐world landscapes. Conserv. Biol. **24**, 1070–1079. (10.1111/j.1523-1739.2009.01440.x) 20136873

[48] Carlo TA, Morales JM. 2016 Generalist birds promote tropical forest regeneration and increase plant diversity via rare‐biased seed dispersal. Ecology **97**, 1819–1831. (10.1890/15-2147.1) 27859154

[49] Camargo PHSA, Pizo MA, Brancalion PHS, Carlo TA. 2020 Fruit traits of pioneer trees structure seed dispersal across distances on tropical deforested landscapes: implications for restoration. J. Appl. Ecol. **57**, 2329–2339. (10.1111/1365-2664.13697)

[50] Dehling DM *et al*. 2020 Similar composition of functional roles in Andean seed‐dispersal networks, despite high species and interaction turnover. Ecology **101**, e03028. (10.1002/ecy.3028) 32112402

[51] Blüthgen N, Menzel F, Blüthgen N. 2006 Measuring specialization in species interaction networks. BMC Ecol. **6**, 9. (10.1186/1472-6785-6-9) 16907983 PMC1570337

[52] Blüthgen N, Menzel F, Hovestadt T, Fiala B, Blüthgen N. 2007 Specialization, constraints, and conflicting interests in mutualistic networks. Curr. Biol. **17**, 341–346. (10.1016/j.cub.2006.12.039) 17275300

[53] Jordano P, Bascompte J, Olesen JM. 2003 Invariant properties in coevolutionary networks of plant–animal interactions. Ecol. Lett. **6**, 69–81. (10.1046/j.1461-0248.2003.00403.x)

[54] Westcott DA, Graham DL. 2000 Patterns of movement and seed dispersal of a tropical frugivore. Oecologia **122**, 249–257. (10.1007/pl00008853) 28308379

[55] Moles AT, Ackerly DD, Webb CO, Tweddle JC, Dickie JB, Westoby M. 2005 A brief history of seed size. Science **307**, 576–580. (10.1126/science.1104863) 15681384

[56] Jost L. 2006 Entropy and diversity. Oikos **113**, 363–375. (10.1111/j.2006.0030-1299.14714.x)

[57] Carlo TA, Yang S. 2011 Network models of frugivory and seed dispersal: challenges and opportunities. Acta Oecologica **37**, 619–624. (10.1016/j.actao.2011.08.001)

[58] García D, Donoso I, Rodríguez‐Pérez J. 2018 Frugivore biodiversity and complementarity in interaction networks enhance landscape‐scale seed dispersal function. Funct. Ecol. **32**, 2742–2752. (10.1111/1365-2435.13213)

[59] Leibold MA *et al*. 2004 The metacommunity concept: a framework for multi‐scale community ecology. Ecol. Lett. **7**, 601–613. (10.1111/j.1461-0248.2004.00608.x)

[60] Carlo TA, Messeder JVS, Allbee SA, Cruz-Mendoza AC, Velázquez SG, Andrzejewski CM, Jenkins TJ, Cordeiro NJ. 2024 Revisiting ecological specialization: the case of plant–frugivore interactions. Oikos e10948. (10.1111/oik.10948)

[61] Dalsgaard B *et al*. 2017 Opposed latitudinal patterns of network‐derived and dietary specialization in avian plant–frugivore interaction systems. Ecography **40**, 1395–1401. (10.1111/ecog.02604)

[62] Dugger PJ *et al*. 2019 Seed‐dispersal networks are more specialized in the neotropics than in the Afrotropics. Glob. Ecol. Biogeogr. **28**, 248–261. (10.1111/geb.12833)

[63] Menke S, Böhning‐Gaese K, Schleuning M. 2012 Plant–frugivore networks are less specialized and more robust at forest–farmland edges than in the interior of a tropical forest. Oikos **121**, 1553–1566. (10.1111/j.1600-0706.2011.20210.x)

[64] Urban D, Keitt T. 2001 Landscape connectivity: a graph-theoretic perspective. Ecology **82**, 1205–1218. (10.1890/0012-9658(2001)082[1205:lcagtp]2.0.co;2)

[65] Lorenzon PC, Massi KG. 2023 The influence of size and distance of Atlantic forest patches on seed rain over tropical pasture. Trop. Ecol. **64**, 193–197. (10.1007/s42965-022-00266-6)

[66] Rehm E, Fricke E, Bender J, Savidge J, Rogers H. 2019 Animal movement drives variation in seed dispersal distance in a plant–animal network. Proc. R. Soc. B **286**, 20182007. (10.1098/rspb.2018.2007) PMC6367185 30963874

[67] Nathan R. 2006 Long-distance dispersal of plants. Science **313**, 786–788. (10.1126/science.1124975) 16902126

[68] Emer C, Galetti M, Pizo MA, Guimarães PR, Moraes S, Piratelli A, Jordano P. 2018 Seed‐dispersal interactions in fragmented landscapes – a metanetwork approach. Ecol. Lett. **21**, 484–493. (10.1111/ele.12909) 29368364

[69] Morán-López T, Rodríguez-Pérez J, Donoso I, Martínez D, Manuel Morales J, García D. 2023 Forest recovery through applied nucleation: effects of tree islet size and disperser mobility on tree recruitment in a temperate landscape. For. Ecol. Manag. **550**, 121508. (10.1016/j.foreco.2023.121508)

[70] Mulwa RK, Böhning‐Gaese K, Schleuning M. 2012 High bird species diversity in structurally heterogeneous farmland in Western Kenya. Biotropica **44**, 801–809. (10.1111/j.1744-7429.2012.00877.x)

[71] Nathan R, Muller-Landau HC. 2000 Spatial patterns of seed dispersal, their determinants and consequences for recruitment. Trends Ecol. Evol. **15**, 278–285. (10.1016/s0169-5347(00)01874-7) 10856948

[72] Côrtes MC, Uriarte M. 2013 Integrating frugivory and animal movement: a review of the evidence and implications for scaling seed dispersal. Biol. Rev. Camb. Philos. Soc. **88**, 255–272. (10.1111/j.1469-185X.2012.00250.x) 23136896

[73] Martínez D, García D. 2015 Disentangling habitat use by frugivorous birds: constant interactive effects of forest cover and fruit availability. Basic Appl. Ecol. **16**, 460–468. (10.1016/j.baae.2015.04.012)

[74] Blendinger PG, Giannini NP, Zampini IC, Ordoñez R, Torres S, Sayago JE, Ruggera RA, Isla MI. 2015 Nutrients in fruits as determinants of resource tracking by birds. Ibis **157**, 480–495. (10.1111/ibi.12274)

[75] Campagnoli M, Christianini A, Peralta G. 2025 Plant and frugivore species characteristics drive frugivore contributions to seed dispersal effectiveness in a hyperdiverse community. Funct. Ecol. **39**, 238–253. (10.1111/1365-2435.14697)

[76] Gleditsch JM, Hruska AM, Foster JT. 2017 Connecting resource tracking by frugivores to temporal variation in seed dispersal networks. Front. Ecol. Evol **5**, 1–11. (10.3389/fevo.2017.00098)

[77] Mason DS, Baruzzi C, Lashley MA. 2022 Passive directed dispersal of plants by animals. Biol. Rev. **97**, 1908–1929. (10.1111/brv.12875) 35770842

[78] Benayas JMR, Bullock JM, Newton AC. 2008 Creating woodland islets to reconcile ecological restoration, conservation, and agricultural land use. Front. Ecol. Environ. **6**, 329–336. (10.1890/070057)

[79] Brancalion PHS, Holl KD. 2020 Guidance for successful tree planting initiatives. J. Appl. Ecol. **57**, 2349–2361. (10.1111/1365-2664.13725)

[80] Paterno GB *et al*. 2024 Diverse and larger tree islands promote native tree diversity in oil palm landscapes. Science **386**, 795–802. (10.1126/science.ado1629) 39541447

[81] Messeder JVS, Guerra TJ, Dáttilo W, Silveira FAO. 2020 Searching for keystone plant resources in fruit‐frugivore interaction networks across the Neotropics. Biotropica **52**, 857–870. (10.1111/btp.12804)

[82] Messeder JVS, Silveira FAO, Cornelissen TG, Fuzessy LF, Guerra TJ. 2021 Frugivory and seed dispersal in a hyperdiverse plant clade and its role as a keystone resource for the neotropical fauna. Ann. Bot. **127**, 577–595. (10.1093/aob/mcaa189) 33151331 PMC8052926

[83] Bechara FC, Dickens SJ, Farrer EC, Larios L, Spotswood EN, Mariotte P, Suding KN. 2016 Neotropical rainforest restoration: comparing passive, plantation and nucleation approaches. Biodivers. Conserv. **25**, 2021–2034. (10.1007/s10531-016-1186-7)

[84] Ramos-Robles M, Dáttilo W, Díaz-Castelazo C, Andresen E. 2018 Fruit traits and temporal abundance shape plant-frugivore interaction networks in a seasonal tropical forest. Sci. Nat. **105**, 29. (10.1007/s00114-018-1556-y) 29610984

[85] Emer C, Jordano P, Pizo MA, Ribeiro MC, da Silva FR, Galetti M. 2020 Seed dispersal networks in tropical forest fragments: area effects, remnant species, and interaction diversity. Biotropica **52**, 81–89. (10.1111/btp.12738)

[86] Fink RD, Lindell CA, Morrison EB, Zahawi RA, Holl KD. 2009 Patch size and tree species influence the number and duration of bird visits in forest restoration plots in Southern Costa Rica. Restor. Ecol. **17**, 479–486. (10.1111/j.1526-100x.2008.00383.x)

[87] Crouzeilles R, Curran M. 2016 Which landscape size best predicts the influence of forest cover on restoration success? A global meta‐analysis on the scale of effect. J. Appl. Ecol. **53**, 440–448. (10.1111/1365-2664.12590)

[88] Munguía-Rosas MA, Montiel S. 2014 Patch size and isolation predict plant species density in a naturally fragmented forest. PLoS One **9**, e111742. (10.1371/journal.pone.0111742) 25347818 PMC4210240

[89] Arteaga LL, Aguirre LF, Moya MI. 2006 Seed rain produced by bats and birds in forest islands in a neotropical Savanna. Biotropica **38**, 718–724. (10.1111/j.1744-7429.2006.00208.x)

[90] Regolin AL, Muylaert RL, Crestani AC, Dáttilo W, Ribeiro MC. 2021 Seed dispersal by Neotropical bats in human-disturbed landscapes. Wildl. Res. **48**, 1–6. (10.1071/WR19138)

[91] Kattge J, Bönisch G, Díaz S, Lavorel S, Prentice IC, Leadley P. 2020 TRY plant trait database – enhanced coverage and open access. Glob. Chang. Biol. **26**, 119–188. 31891233 10.1111/gcb.14904

[92] Tobias JA *et al*. 2022 AVONET: morphological, ecological and geographical data for all birds. Ecol. Lett. **25**, 581–597. (10.1111/ele.13898) 35199922

[93] Nowak L *et al*. 2022 Avian seed dispersal may be insufficient for plants to track future temperature change on tropical mountains. Dryad Digital Repository (10.5061/dryad.4f4qrfjdm)

[94] Landim AR. 2025 Data and code used in the paper: Functional connectivity of animal-dispersed plant communities depends on the interacting effects of network specialization and resource diversity. Zenodo (10.5281/zenodo.14793841) PMC11881642 40042259

[95] Landim AR, Neuschulz EL, Donoso I, Sorensen M, Mueller T, Schleuning M. 2025 Supplementary material from: Functional connectivity of animal-dispersed plant communities depends on the interacting effects of network specialization and resource diversity. Figshare (10.6084/m9.figshare.c.7682104) PMC11881642 40042259

